# Disparities in greenspace associated with sleep duration among adolescent children in Southern California

**DOI:** 10.1097/EE9.0000000000000264

**Published:** 2023-08-01

**Authors:** Charlie Zhong, Xiaozhe Yin, Masoud Fallah-Shorshani, Talat Islam, Rob McConnell, Scott Fruin, Meredith Franklin

**Affiliations:** a Department of Population and Public Health Sciences, Keck School of Medicine, University of Southern California, Los Angeles, California; b Department of Statistical Sciences and School of the Environment, University of Toronto, Toronto, Ontario, Canada

**Keywords:** Artificial light at night, Children, Circadian rhythm, Epidemiology, Green space, Sleep disruption

## Abstract

More than half of adolescent children do not get the recommended 8 hours of sleep necessary for optimal growth and development. In adults, several studies have evaluated effects of urban stressors including lack of greenspace, air pollution, noise, nighttime light, and psychosocial stress on sleep duration. Little is known about these effects in adolescents, however, it is known that these exposures vary by socioeconomic status (SES). We evaluated the association between several environmental exposures and sleep in adolescent children in Southern California.

**Methods:**

In 2010, a total of 1476 Southern California Children’s Health Study (CHS) participants in grades 9 and 10 (mean age, 13.4 years; SD, 0.6) completed a questionnaire including topics on sleep and psychosocial stress. Exposures to greenspace, artificial light at night (ALAN), nighttime noise, and air pollution were estimated at each child’s residential address, and SES was characterized by maternal education. Odds ratios and 95% confidence intervals (95% CIs) for sleep outcomes were estimated by environmental exposure, adjusting for age, sex, race/ethnicity, home secondhand smoke, and SES.

**Results:**

An interquartile range (IQR) increase in greenspace decreased the odds of not sleeping at least 8 hours (odds ratio [OR], 0.86 [95% CI, 0.71, 1.05]). This association was significantly protective in low SES participants (OR, 0.77 [95% CI, 0.60, 0.98]) but not for those with high SES (OR, 1.16 [95%CI, 0.80, 1.70]), interaction *P* = 0.03. Stress mediated 18.4% of the association among low SES participants.

**Conclusions:**

Residing in urban neighborhoods of greater greenness was associated with improved sleep duration among children of low SES but not higher SES. These findings support the importance of widely reported disparities in exposure and access to greenspace in socioeconomically disadvantaged populations.

**What this study adds**

In contrast to previous studies in adults, we did not find a strong association between artificial light at night, noise, and air pollution and sleep outcomes among adolescents. We did observe a protective association with greenspace and sleep duration. Several recent studies have highlighted the interaction between greenspace and health outcomes related to socioeconomic status, which we observed in our study as well. When stratified by socioeconomic status, the protective association with greenspace was only observed among the low socioeconomic participants, while noise was associated with reduced sleep duration in high socioeconomic participants.

## Background

The Centers for Disease Control and Prevention (CDC) recognize sleep disorders as a public health concern; with only an estimated one-third of adults in the United States getting the recommended minimum of 7 hours of sleep each night.^1,2^ As children and adolescents need more sleep for healthy growth and development, it is recommended that adolescents get at least 8 hours of sleep each night. However, data from the United States National Health and Nutrition Examination Survey (NHANES) show that over half of the adolescents aged 16 to 19 years old do not meet this recommendation.^3^ Sleep disruptions increase during this time as adolescents face increased academic and social burdens when entering high school.^4^ Disruptions to circadian rhythm due to early school start times are the most impactful in adolescents,^5^ and stress can lead to activation of the hypothalamic-pituitary-adrenal axis, leading to neuroendocrine disruptions of sleep regulation. Insufficient sleep can also lead to increased stress and poorer academic performance,^6,7^ the effects of which have been found to be greater among adolescents whose parents have less formal education.^8^ Across the lifespan, insufficient sleep has also been associated with increased morbidity and mortality.^9^

Urban environmental exposures are becoming recognized as another important factor that can have an influence on sleep.^10,11^ For example, residing in a noisier neighborhood has been associated with later sleep onset and shorter sleep duration.^10^ Air pollution has been associated with both obstructive sleep apnea^12^ and sleep-disordered breathing,^13^ and artificial light at night (ALAN) can disrupt natural circadian rhythm, delaying onset of sleep, affecting duration,^14^ and increasing the risk of sleep disorders.^15^

In contrast, greenspace can improve one’s perception of their surroundings and has been shown to reduce stress^16,17^ and improve sleep.^18,19^ Greenspace can also partially attenuate the detrimental impacts of noise and air pollution.^20–22^ However, these environmental exposures are not equally distributed among different populations, as minority and low-income individuals are more likely to reside in areas of greater noise, air pollution, ALAN, and lower levels of greenspace.^23–25^

While some studies have evaluated environment or stress and adolescent sleep patterns separately, none have assessed them together. We previously reported that sleep duration partially mediated associations between greenspace, ALAN, and stress in adolescents.^26^ Here, we focused on environmental factors that directly associate with sleep; we also examined the role of stress.

## Methods

### Study population and outcomes

The Children’s Health Study (CHS) comprises several cohorts of children in Southern California recruited at different time points. Our analysis focused on the final CHS cohort, which was initiated in 2003 and followed children in eight communities (Anaheim, Glendora, Long Beach, Mira Loma, Riverside, Santa Barbara, San Dimas, and Upland) until 2012.^27^ In 2010, when study participants were in grades 9 and 10, a questionnaire was administered that included questions about sleep and stress. A total of 1549 adolescents responded to the questionnaire. After excluding those who we were unable to assign residential exposures to, we were left with an analytic cohort of 1476. Sleep outcomes were assessed from three questions ascertaining: sleep duration (<5 hours to >11 hours in 7, 1-hour long categories), trouble going to sleep (yes, no, and sometimes), and trouble staying asleep (yes, no, and sometimes). All responses were dichotomized: less than 8 hours or 8 hours or more (based on the recommendations from the American Academy of Sleep Medicine^28^), and any versus no for trouble going to sleep or staying asleep. Stress was assessed with the 4-item version of the Perceived Stress Scale (PSS).^29^ A composite stress score ranging from 0 to 16 was calculated as the sum of the 4 responses (higher scores indicated greater perceived stress). Additional covariates included race/ethnicity, sex, and socioeconomic status (SES) based on the highest level of maternal education.^30^ Study participants’ residential addresses were geocoded for spatial linkages with environmental exposure data. Parents provided informed consent and children provided assent; study protocols were approved by the Institutional Review Board at the University of Southern California. A description of the cohort has been previously published.^27^

### Environmental exposures

Greenspace was assessed with a 250-meter gridded enhanced vegetation index (EVI) derived from imagery obtained by the Moderate Resolution Imaging Spectroradiometer (MODIS) instrument onboard NASA’s Earth Observing System Terra Aqua Satellite. EVI corresponds to the ratio of near infrared to visible light and ranges from a minimum of −1 (completely barren land) to a maximum of 1 (high-density vegetation). Based on 2-week observations, an annual 2010 average EVI was assigned to each participant based on the grid in which they resided.

ALAN was assessed with the New World Atlas of Artificial Night Sky Brightness.^31^ The New World Atlas is an evolution of the previous World Atlas and estimates a global 750-meter gridded measure of light at the zenith (the sky directly overhead) in millicandela per meter squared (mcd/m^2^) based on the observations from the Visible Infrared Imaging Radiometer Suite satellite during the latter half of 2014, combined with thousands of handheld sky quality measurements. This combination has been shown to produce better estimates of exposure to light than satellite data by itself.^32^

Noise was estimated using the Computer Aided Noise Abatement (CadnaA) model, a widely-used commercial software package estimating road traffic noise. Within CadnaA we used the German noise emission standard RLS-90, which estimates the A-weighted (human perception matched) equivalent average sound energy (LAeq) in decibels (dB), based on the road network, number of vehicles per hour, percentage of heavy vehicles, speeds of passenger car and heavy vehicles, road gradients, road surface types, and any multiple sound reflections from buildings. While much of the CadnaA inputs were static (e.g., road network and speed limits), traffic volumes were reflective of the 2018 conditions in LA County. The noise level of each grid was calculated by incorporating distance to the sources (road traffic) and obstacles (buildings). From CadnaA we estimated average daytime and nighttime noise separately for each study participant’s residential address. Model LAeq estimates were validated against measurements taken in 2019, showing excellent agreement.^33^ As the traffic volumes in LA county did not change significantly between 2010 and 2018,^34^ we believe they are a good representation of the noise levels at the time of CHS data collection.

Ambient near-roadway air pollution was estimated using the CALINE4 traffic line source dispersion model.^35^ The model uses roadway geometry, vehicle traffic volumes, vehicle emission factors, and meteorological conditions to estimate pollutant exposure as a function of distance and direction from roadway. This model has been shown to be a good predictor of NOx in Southern California.^36^ Annual 2010 average concentrations of freeway- and non-freeway-related NOx were assigned to each participant’s address.

Regional air pollution was assessed with a 4.4-kilometer gridded estimate of concentrations of particulate matter less than 2.5 micrometers in diameter (PM_2.5_) derived from the Multi-Angle Imaging SpectroRadiometer (MISR) instrument onboard NASA’s Terra satellite. Estimates based on the MISR aerosol optical depth correlated well (R = 0.71) with PM_2.5_ measurements from ground monitoring stations in Southern California.^37^ Average air pollutant concentrations for 2010 were assigned to each participant’s address.

### Statistical methods

We evaluated cross-sectional associations between environmental exposures (greenspace, ALAN, noise, and air pollution) and self-reported sleep duration, sleep latency (time to fall asleep), and trouble staying asleep using logistic regression, calculating odds ratios (ORs) and 95% confidence intervals (95% CI). We mutually adjusted for all environmental exposures and a priori covariates, race/ethnicity (Asian, Black, Hispanic, Mixed, White, or Unknown/Missing), sex (male vs. female), age, SES (maternal education less than college vs. college graduate), and CHS community.^7,10,13,18,38,39^ Associations were reported per interquartile range (IQR) change in exposure. Because these environmental exposures were previously associated with stress in our study population,^26^ we assessed the mediating role of self-reported stress on our exposure-sleep associations and report it as a percent.^40^ Due to possible interactions between environmental exposures and SES,^41,42^ we assessed interaction between each exposure and SES among those that reported maternal education, further stratifying for those that were statistically significant (*P*-value < 0.05). All analyses were conducted in the R Language, version 4.1.0 (R Foundation for Statistical Computing, Vienna, Austria).

## Results

A total of 1476 children responded to the 2010 questionnaire and had available environmental exposures. The average age of participants was 13.4 and most (64%) reported sleeping at least 8 hours (Table 1). Community-specific distributions of the environmental exposures for study participants are provided in Supplemental Figure 1; http://links.lww.com/EE/A232. Compared with participants who reported sleeping at least 8 hours, those who reported sleeping less than 8 hours had higher levels of all environmental exposures except for greenspace, which was higher in the participants that slept 8 hours or more (Table 1). The average PSS-4 score was 5.2 among those who slept 8 hours or more and 6.8 among those who did not.

**Table 1.** Distribution of demographic and environmental exposures in the Children’s Healthy Study.

	Sleep duration (hours)			
	≥8 hours		<8 hours	
	N	%	N	%
Sex				
Male	469	49.5%	240	45.4%
Female	478	50.5%	289	54.6%
Age (years; SD)	13.42	0.6	13.48	0.63
Race/ethnicity				
Asian	39	4.1%	28	5.3%
Black	13	1.4%	10	1.9%
Hispanic	500	52.8%	312	59.0%
Mixed	48	5.1%	29	5.5%
Unknown/missing	20	2.1%	14	2.6%
White	327	34.5%	136	25.7%
Maternal education				
Less than college	584	61.7%	378	71.5%
College graduate	294	31.0%	119	22.5%
Missing	69	7.3%	32	6.0%
Community (row %)				
Anaheim	79	53.7%	68	46.3%
Glendora	179	71.9%	70	28.1%
Long Beach	51	54.3%	43	45.7%
Mira Loma	114	55.3%	92	44.7%
Riverside	96	64.4%	53	35.6%
Santa Barbara	153	72.9%	57	27.1%
San Dimas	133	63.6%	76	36.4%
Upland	142	67.0%	70	33.0%
Exposed to secondhand smoke	69	7.3%	56	10.6%
Stress (PSS-4 score; SD)	5.2	3	6.8	3
Exposures, median (IQR)				
Greenspace (EVI)	0.21	0.18–0.24	0.20	0.17–0.22
Light at night (mcd/m^2^)	3.5	2.5–4.1	3.7	3.1–4.6
Nighttime noise (dB)	45.7	41.0–51.5	47	41.2–46.8
Freeway NOx (ppb)	7.6	4.0–14.9	8.8	5.5–14.4
Regional PM_2.5_ (μg/m^3^)	14.4	14.0–15.7	14.8	14.2–16.2

In univariate (single exposure) analyses adjusted only for CHS community, both ALAN (OR, 1.44 [95% CI, 1.07, 1.94]) and greenspace (OR, 0.78 [95% CI, 0.66, 0.93]) were significantly associated with sleep duration (Supplemental Table 2; http://links.lww.com/EE/A232). However, in the multiexposure models adjusting for all confounders, there was an attenuation of the environmental exposures, and ALAN (OR, 1.29 [95% CI, 0.90, 1.86]) and greenspace (OR, 0.86 [95% CI, 0.71, 1.05]) were no longer significantly associated with sleep duration (Table 2). Regional air pollution, freeway-related air pollution, and noise were not associated with sleep duration. We also did not observe any significant association between environmental exposures and sleep latency or trouble staying asleep (Supplemental Tables 3 and 4; http://links.lww.com/EE/A232).

**Table 2.** Multivariate association between sleeping less than 8 hours nightly and environmental and other exposures in the Children’s Health Study.

	Main analyses*		Further adjusted for stress*		
	OR	95% CI	OR	95% CI	% mediated
Environmental Exposure (IQR)					
Greenspace (0.058 EVI)	0.86	(0.71, 1.05)	0.89	(0.73, 1.08)	22.1%
Light at night (1.64 mcd/m^2^)	1.29	(0.90, 1.86)	1.29	(0.89, 1.88)	10.8%
Nighttime noise (10.6 dB)	0.97	(0.79, 1.19)	0.97	(0.79, 1.19)	0.5%
Freeway NOx (10.5 ppb)	0.97	(0.87, 1.08)	0.96	(0.86, 1.07)	4.9%
Regional PM_2.5_ (1.80 μg/m^3^)	0.95	(0.64, 1.39)	0.87	(0.59, 1.30)	9.2%
Maternal education					
Less than college	Reference		Reference		
College graduate	0.77	(0.57, 1.03)	0.76	(0.56, 1.02)	
Sex					
Female	Reference		Reference		
Male	0.89	(0.71, 1.12)	0.97	(0.77, 1.24)	
Age	1.23	(1.02, 1.49)	1.24	(1.02, 1.51)	
Exposed to secondhand smoke					
No	Reference		Reference		
Yes	1.25	(0.84, 1.85)	1.38	(0.76, 2.51)	
Stress			1.14	(1.11, 1.20)	

*Odds ratio reported per IQR and represent odds of sleeping less than the recommended 8 hours each night, adjusted for variables listed, race/ethnicity, and CHS community.

Other nonenvironmental factors associated with reduced sleep included being older, lower maternal education, and higher stress (Table 2). Older children had higher odds of not sleeping at least 8 hours (OR, 1.23 [95% CI, 1.02, 1.49]), while higher maternal education was suggestively protective with lower odds of not sleeping at least 8 hours (OR, 0.77 [95% CI, 0.57, 1.03]). Stress was significantly associated with reduced sleep duration with 1.14 increased odds (95% CI, 1.11, 1.20) of sleeping less than 8 hours.

In assessing mediation, only greenspace was statistically significantly associated with lower stress (beta = −0.31; *P*-value 0.03; Supplemental Table 1; http://links.lww.com/EE/A232) and stress mediated 22% of the association between sleep duration and greenspace. When assessing effect modification of the environmental exposures by maternal education, there was only a statistically significant interaction between greenspace and maternal education (Supplemental Figure 2; http://links.lww.com/EE/A232, Supplemental Table 5; http://links.lww.com/EE/A232). When stratified, greenspace was significantly protective of sleep duration in participants who reported low maternal education (OR, 0.77 [95% CI, 0.60, 0.98]; Table 3). Similar to the overall analysis, stress mediated 18% of the association with greenspace in this population but was not associated with sleep duration among adolescents in households of high maternal education. We did not observe significant exposure-mediator interaction or strong violation of the sequential ignorability assumption in our mediation models (ρ = 0.2).^43^ Compared with the low maternal education population, those with high maternal education had lower levels of ALAN, nighttime noise, freeway NOx, PM_2.5_, and higher levels of greenspace (Supplemental Table 5; http://links.lww.com/EE/A232). An IQR increase in nighttime noise was associated with 1.69 increased odds (95% CI, 1.09, 2.65) of not sleeping 8 hours in the high maternal education participants, and exposure to secondhand smoke in high maternal education households was associated with 3.79 increased odds (95% CI, 1.31, 10.93) of not sleeping 8 hours.

**Table 3.** Multivariate association between environmental exposures and sleep duration in the Children’s Health Study stratified by maternal education.

	Low maternal education					High maternal education				
	Main analyses*		Further adjusted for stress*		% mediated	Main analyses*		Further adjusted for stress*		% mediated
	OR	95% CI	OR	95% CI		OR	95% CI	OR	95% CI	
Environmental exposure (IQR)										
Greenspace (0.058 EVI)	0.77	(0.60, 0.98)	0.81	(0.63, 1.03)	18.4%	1.16	(0.80, 1.70)	1.14	(0.78, 1.68)	1.2%
Light at night (1.64 mcd/m^2^)	1.28	(0.83, 1.96)	1.29	(0.83, 1.99)	6.2%	1.59	(0.72, 3.51)	1.53	(0.68, 3.46)	9.6%
Nighttime noise (10.6 dB)	0.83	(0.65, 1.06)	0.81	(0.64, 1.04)	3.1%	1.69	(1.09, 2.65)	1.79	(1.13, 2.84)	0.3%
Freeway NOx (10.5 ppb)	1.04	(0.91, 1.18)	1.04	(0.91, 1.19)	0.1%	0.75	(0.50, 1.13)	0.71	(0.46, 1.08)	10.7%
Regional PM_2.5_ (1.80 μg/m^3^)	0.98	(0.61, 1.57)	0.92	(0.57, 1.49)	1.5%	0.77	(0.36, 1.66)	0.69	(0.31, 1.53)	12.3%
Sex										
Female	Reference		Reference			Reference		Reference		
Male	0.90	(0.69, 1.18)	0.97	(0.74, 1.28)		0.76	(0.48, 1.22)	0.87	(0.53, 1.41)	
Age	1.14	(0.91, 1.44)	1.17	(0.93, 1.47)		1.53	(1.05, 2.23)	1.45	(0.98, 2.14)	
Exposed to secondhand smoke										
No	Reference		Reference			Reference		Reference		
Yes	1.65	(0.68, 3.98)	1.59	(0.65, 3.91)		3.79	(1.31, 10.93)	2.36	(0.78, 7.21)	
Stress			1.13	(1.08, 1.19)				1.17	(1.08, 1.27)	

*Odds ratio reported per IQR and represent odds of sleeping less than the recommended 8 hours each night, adjusted for variables listed, race/ethnicity, and CHS community.

## Discussion

Adolescent children in low SES families residing in areas of greater greenspace were more likely to sleep the recommended 8 hours each night, and the protective effect of greenspace on sleep duration was similar to those observed in adult populations.^18,44^ This association was moderately mediated by stress. Greenspace can partially attenuate levels of environmental pollutants such as air pollution by removing particulates,^45^ and trees can reduce noise pollution by disrupting the propagation of sound waves.^46^ The attenuation of the effect of noise due to greenspace may be due to the extent of tree cover and possibly their configuration in the landscape.^46,47^ While EVI improves upon NDVI by correcting for vegetation density and adjusting for features related to tree canopies, they are both unitless measures that only capture the relative amount of greenspace.^48^ Despite the greater specificity of EVI, we are limited by its spatial resolution in terms of our ability to evaluate effects at the residence level. A better understanding of which specific factors related to greenspace, such as tree density or access to parks, are associated with improved outcomes could provide clues as to how urban greenspaces can be tailored to benefit public health. Beyond physical alterations to the environment, greenspace may also influence how we perceive our surroundings and offset or reduce the stress from exposures such as noise,^49^ which has been associated with improved mental health and cognitive development in children.^50,51^ Greenspace was the only environmental exposure significantly associated with decreased self-reported stress in our participants (Supplemental Table 1; http://links.lww.com/EE/A232) and stress appeared to partially mediate the protective effect of greenspace on sleep duration.

In univariate exposure models, greenspace (OR, 0.78 [95% CI, 0.66, 0.93)] and ALAN (OR, 1.44 [95% CI, 1.07, 1.94]) were associated with decreased sleep duration (Supplemental Table 2; http://links.lww.com/EE/A232). However, ALAN was no longer significant in the multiexposure model. ALAN and greenspace were strongly, negatively correlated in our cohort (σ = −0.60) and may contribute to difficulty identifying associations.

There did appear to be differing risk factors between the two SES strata. When stratified by maternal education, as a marker of SES, the benefit of greenspace was significantly protective in the low SES group (OR_low SES_ 0.77 [95% CI, 0.06, 0.98] vs. OR_high SES_ 1.16 [95% CI, 0.80, 1.70]; Table 3). This is consistent with other findings suggesting that lower SES may benefit more from an increase in greenspace than those of higher SES.^41^ EVI was higher (as well as lower air pollution, ALAN, and noise) among the high SES group compared with the low SES group (Supplemental Table 6; http://links.lww.com/EE/A232). Large roadways, a major contributor to air and noise pollution are more likely to pass through low-income neighborhoods,^52^ and distance to and number of parks is lower in these communities as well.^53^ It is believed that these disparities in exposures, in part, contribute to the effect modification seen with greenspace as lower SES households have lower vehicle ownership and are more likely to rely on their surrounding environment.^41^ Low SES individuals are exposed to more stressors,^54^ which may be why stress was a stronger mediator of greenspace in this population, although both low SES and high SES adolescents in our study who did not sleep at least 8 hours reported similar levels of stress (PSS score of 6.5 vs. 6.3; *P*-value = 0.44). These findings support the widely reported inequities in exposure to environmental pollutants and greenspace observed in minority and socioeconomically disadvantaged populations.^23–25^

In contrast to studies in adult populations,^14,44,55^ we did not observe a significant association between ALAN and any of our sleep outcomes in adolescents in our multivariate models. In a large cohort of Californian teachers, a 5 mcd/m^2^ increase in ALAN was associated with 1.13 (95% CI, 1.07, 1.20) increased odds of sleeping less than 7 hours,^44^ the recommended length for adults, compared with the 1.29 increased odds (95% CI, 0.90, 1.86 per IQR) observed in our study. Because this adult cohort was not limited to the Southern California area, the average ALAN was lower (2.81 vs. 3.6). We did not differentiate between weekday and weekend sleep, and ALAN was shown to be associated with increased weekend sleep latency in a study of US adolescent children by Paksarian et al.^56^ Light stimuli at night can disrupt melatonin levels, thereby delaying onset of sleep.^14,57^ Daytime exposure to natural light also impacts sleep duration and latency,^58^ and adults are more likely to spend their days indoors while at work, and exposure to indoor lighting may not provide the necessary stimulus for proper circadian rhythms.^59^ In addition to melatonin, our bodies have a physiological inclination to falling asleep at certain times, known as chronotype, commonly referred to as being a “morning” or “evening” person. Adolescents are at the age range when chronotype is the latest during life^5^ and this late chronotype appears to have more impact on sleep than onset of melatonin in adolescents.^60^

While we did not observe a significant overall association with noise, it appeared to increase risk of not sleeping 8 hours in the high SES participants (OR, 1.79 [95% CI, 1.13, 2.84]; Table 3). These results are similar to those reported by Mayne et al. who observed increased risk of reduced sleep duration in adolescents in a higher income population (Supplemental Table 6; http://links.lww.com/EE/A232).^61^ While the noise levels were lower, there was a wider distribution of noise among these participants compared with the low SES participants, which may have contributed to the ability to observe an association. The association with sleep duration and noise in low SES participants was inverted (OR, 0.83 [95% CI, 0.65, 1.06]), which may have also contributed to the null overall findings. Traffic-based noise models are less accurate in residential neighborhoods where there is less accurate information about traffic patterns on smaller streets. These noise models also estimate daytime noise and apply a penalization to generate nighttime levels, which may not necessarily result in the most representative nighttime exposures.

Strengths of our study include evaluation of several validated geospatial estimates for our environmental exposures in a single model.^31,37,62^ However, our lower resolution estimates of environmental exposures showed much more between community variation than within community variation (regional PM_2.5_ and ALAN, Supplemental Figure 1; http://links.lww.com/EE/A232), which may impact our ability to observe associations between sleep and those exposures. The variations in resolution and the relationship (both synergistic and antagonistic) between these mixtures may confound observed associations.^63^ These exposure mixture and patterns are unique to the Southern California region and may not encompass the various complex environmental mixtures we are exposed to. In particular, the distribution of environmental exposures and SES in the United States are not reflective of patterns seen in other countries such as Europe.^64^ We used maternal education as an individual level marker of SES, which has been shown in previous studies to be a robust measure when assessing childhood health.^30,65^ Our measures of sleep were a limitation as they were self-reported and we did not collect any other individual level factors that may impact sleep and circadian rhythm, such as chronotype or mobile device use. While there is evidence that mobile device use disrupts sleep,^66^ we do not believe it is associated with the environmental exposures in our cohort. We also only have a report of overall sleep and did not assess sleep misalignment between schooldays and weekends. Aside from sleep duration, our other sleep domains were assessed subjectively (e.g., for latency we asked whether they had trouble falling asleep instead of time taken to fall asleep). Adolescents tend to exhibit later chronotypes, which conflicts with early school start times, contributing to insufficient sleep, increased stress, and poorer academic performance.^6,7,60^ To address these concerns, in 2019, California was the first state to pass legislation mandating later school start times. Our results are consistent with another report of environmental exposures and sleep among adolescents that used objective measurements.^61^ Our environmental exposures were limited to the outdoor environment, but they have shown correlations with indoor measures.^67,68^ Of particular concern are other sources of indoor light pollution, which have become a greater concern in recent years with the increased prevalence of mobile devices and usage patterns.^66,69^ Finally, we did not evaluate the role of physical activity as it was not well ascertained for this cohort.

## Conclusions

In summary, we observed a protective effect of increased greenspace on sleep duration in adolescent children. This effect appeared to be mediated by stress and associations were stronger in lower SES households. Finer spatial resolution of urban environmental exposures, more complex evaluation of exposure mixtures, and greater subject numbers may be needed to fully understand the complex nature of urban surroundings and how they affect sleep in adolescents.

## ACKNOWLEDGMENTS

We thank Dr. Fabio Falchi, PhD (Istituto di Scienza e Tecnologia dell’Inquinamento Luminoso) for providing data for the New World Atlas of Artificial Night Sky Brightness and Dr. Travis Longcore, PhD (UCLA Institute of the Environment and Sustainability) for his expertise in interpreting artificial light at night exposures.

## Conflicts of interest statement

The authors declare that they have no they have no conflicts of interest with regard to the content of this report.

This work was supported by a National Institute of Environmental Health Sciences of the National Institutes of Health (grants T32ES013678, P30ES007048, and P2C ES033433) and the Health Effects Institute, an organization jointly funded by the US Environmental Protection Agency (assistance award No. CR-83590201) and certain motor vehicle and engine manufacturers.

The data presented in the current study are not publicly available due data confidentiality but are available from the corresponding author on reasonable request.

## Supplementary Material
